# Transition shock among nursing interns and its relationship with patient safety attitudes, professional identity and climate of caring: a cross-sectional study

**DOI:** 10.1186/s12912-024-01722-5

**Published:** 2024-01-24

**Authors:** Sha Zhao, Qinglong Liang, Hong Tao, Sisi Fan, Yuting Xia, Lihong Zeng, Guiyun Wang, Huan Liu, Hui Huang, Jinnan Xiao

**Affiliations:** 1 The Third Xiangya Hospital of Central South University, Changsha, China; 2 Xiangya School of Nursing, Central South University, Changsha, Hunan China; 3 Shandong Xiehe University, Jinan, China

**Keywords:** Nursing interns, Transition shock, Patient safety attitudes, Professional identity, Climate of caring

## Abstract

**Background:**

Nursing interns often experience lots of challenges during their clinical nursing internships, which can adversely affect career decisions and result in a squandering of nursing education resources. Patient safety attitudes, professional identity and climate of caring may affect nursing interns’ clinical experience. However, more evidence is requested to validate these relationships for nursing educators to develop effective education programs and facilitate interns’ successful transition.

**Methods:**

This was a cross-sectional study, which used a convenience sampling method to recruit 387 nursing interns during December 2022 to April 2023 in university affiliated hospital in Hunan province, China. Data were collected using standardized scales. Spearman correlation and multiple regression analysis were employed to examine the relationship between transition shock, patient safety attitudes, professional identity, and climate of caring.

**Results:**

Nursing interns experienced transition shock at a moderate level and the highest levels of transition shock in response to overwhelming practicum workloads, with the second being related to the conflict between theory and practice. Transition shock was negatively correlated with patient safety attitudes, professional identity and climate of caring among nursing interns.

**Conclusions:**

Nursing managers and educators need to value the transition shock experienced by nursing interns. Our study suggests that developing a strong sense of professional identity and a positive attitude toward patient safety can be effective in reducing the level of transition shock among nursing interns. In addition, a caring climate within the nursing unit can significantly enhance the overall experience of nursing interns. This can be achieved by enhancing the support of clinical mentors, providing patient safety-focused education, and facilitating team communication among nurses.

## Introduction

An irreplaceable period in nursing education is the clinical nursing internship [1], it occurring typically during the third or fourth year of nursing education program after nursing students complete their curriculum studies. During the clinical internship phase, nursing students enter the clinical hospital setting to apply the knowledge and practices they have learned in school, providing daily nursing care to patients under the supervision of experienced nurses. This is essential period in transition from being nursing student to registered nurse. In China, nursing students are required to complete more than 8 months of clinical nursing internship before they are registered as nurses [2]. Nursing internship aims to equip the nursing interns with the necessary professional skills, and ensures that they are well-prepared to deliver high-quality care and make a meaningful impact in the healthcare field [3]. However, nursing interns experience considerable challenges during internship due to the gap between theory and practice, high workload, and insufficient clinical knowledge [4], these challenges cause transition shock, which represents the physical and psychological experiences of adjusting to new responsibilities, roles, knowledge and relationships [5, 6]. Transition shock not only increases the difficulty to adapting the professional role of a nurse [7], but also reduces their job satisfaction and engagement [8, 9]. Research indicated that transition shock affect the quality of care of nurses [10], and lead to high turnover rate for nurses [8]. Therefore, it is of great practical significance to understand the current state and correlative factors with regarding nursing interns’ transition shock and to help them lead a successful transition from students to a professional nurse.

Recent literature has indicated a relationship between transition shock and patient safety attitudes, but more evidences are warranted among nursing interns. Patient safety attitudes means the faith that strives to minimize possible harm to patients [11]. Transition shock leaves nursing interns focus on time management and task completion rather than patient safety and personalized care. A cross-sectional study among 176 newly graduated nurses showed higher levels of transition shock were associated with adverse patient events [10]. A qualitative study described that new graduate nurses are often fearful of making mistakes or missing something that could result in the deterioration of patient care [12]. A predictive study among 351 nurses also highlighted that increased patient safety attitudes could reduce missed nursing care and improve healthcare quality [13]. However, current research on patient safety attitudes focused on newly graduated nurses and register nurses, the relationship between interns’ patient safety attitudes and transition shock remains unclear.

Previous research has substantiated the perspective that professional identity may be related to transition shock and patient safety attitudes. Professional identity is a dynamic self-concept that forms the psychological foundation for people to excel in their work, and interns mostly develops it during internship [14, 15]. A cross-sectional study of 474 nursing students found those with higher professional identity values had lower role stress levels [16]. An intervention study found that a nursing professional identity promotion program significantly reduced the incidence of safety events [17]. Unlike register nurses who already have a relatively stable career, nursing interns will decide whether to pursue nursing based on their internship experience. Therefore, it is necessary for nursing educators to understand the association of professional identity, patient safety attitudes with transition shock among nursing interns.

Previous studies mainly focus on identifying individual factors of transition shock [18, 19], such as self-efficacy, resilience and education level, limited attention was paid to organizational factors affecting transition shock among nursing interns. Recently, a few empirical studies suggested a good workplace environment may affect transition shock among nursing interns. Climate of caring incorporates a work environment in which nursing interns can express their stress and anxiety without fear, and where their feelings are considered by the medical team members [20]. Study has suggested that a good climate of caring environment alleviated employees’ emotional exhaustion [21]. Moreover, climate of care has a direct and significant impact on job performance, and role stress [22]. Enhancing nursing students’ perceptions of climate of caring improves their ability to apply professional behaviors [23].However, current studies failed to clearly identify the association between climate of caring among work environment with transition shock of nursing interns.

Therefore, investigating the association between transition shock, patient safety attitudes, professional identity, and climate of caring would yield valuable insights for nursing educators and managers on how to enable successful transitions to practice among nursing interns, providing quality and safe nursing services for patients.

## Methods

### Study design

This study used a descriptive cross-sectional design to determine the relationship between transition shock with patient safety attitudes, professional identity and climate of caring among nursing interns.

### Setting and sample

Convenience sampling was used to recruit nursing interns from a university affiliated hospital in Hunan Province, China, which accommodates around 350–400 nursing interns annually from more than 30 different universities and colleges in Hunan Province. During December 2022 to April 2023, a total of 387 nursing interns completed their internship at this hospital and all of them were invited for eligibility assessment. The eligibility criteria were as follows: (1) either in the third or fourth year of nursing education, known as the clinical internship phase, (2) more than 8 months of clinical internship [2].

### Sample size

The sample size needed for this study was calculated using Kendall’s cross-sectional investigation formula of N × (10 − 15)(N = independent variable and there was a maximum of 26 independent variables include 9 demography variables and 17 subscales ) [24]. With a rate of 20% invalid questionnaires, at least 312 participants were required for this study.

### Data collection

Prior to the study, permission was obtained from the nursing department at the hospital. Online questionnaires were then delivered to nursing interns using an application called “Wenjuanxing (Changsha, China).” Through this application, the questionnaires could be easily completed and submitted through mobile phone. It took approximately 10–15 min to complete the questionnaire. To ensure the quality and completeness of the questionnaire, all questions were compulsory and could only be completed once per IP address to avoid repeat filling. The researcher collected the questionnaires from the backend and eliminate invalid submissions where all answers were identical and submitted within five minutes. Overall, 387 nursing interns were surveyed in this study, with 356 valid questionnaires returned, resulting in a valid return rate of 91.98%.

### Instruments

#### Demographic questionnaire

The questionnaire was designed by the research team to collect demographic information for nursing interns. Demographic variables, including gender, age, education level, the reason for choosing a nursing major, family connections to nursing, body health situation, whether like nursing professional.

#### Transition shock of undergraduate nursing students scale

Transition shock was measured using the Transition Shock Scale for Undergraduate Nursing Students, developed by Kim [25]. The scale consists of 18 items scored on a 4-point (ranging from 1 = totally disagree to 4 = totally agree). It is grouped into 6 subscales: conflict between theory and practice (3 items), overwhelming practicum workload (3 items), loss of social support (2 items), shrinking interpersonal relationship (3 items), confusion in professional nursing values (5 items), incongruity in clinical practicum and personal lives (2 items). Higher scores indicated a stronger transition shock [26]. The scale was translated into Chinese and applied to evaluate transition shock of nursing interns, showing a good validity in China [27], the Cronbach alpha coefficient of the overall scale was 0.912, in this study was 0.901.

#### Patient safety attitudes and professional qualities of trainee nursing students scale

This scale has comprised six dimensions divided into two parts, four subscales were employed in this study to assess nursing interns’ patient safety attitudes [11, 28]. These 4 subscales include: team culture (6 items), safety culture (8 items), error reporting culture (5 items), intention to implement safety behavior (4 items). The items are evaluated on a 5-point scale ranging from 1 (strongly disagree) to 5 (strongly agree) and NA (not application) scored zero, with higher scores indicating better patient’s safety attitudes [29]. The reliability for these four dimensions in this study ranged from 0.845 to 0.965, with a Cronbach’s α of 0.954 for the overall subscales. The Kaiser–Meyer–Olkin (KMO) value was 0.943 (Bartlett’s *P* < 0.01). These results shown that the four subscales are both reliable and valid for assessing nursing students’ patient safety attitudes in this study.

#### Professional identification scale

Nursing interns’ professional identity level was measured by a professional identification scale, which was developed by Brown [30] and Hong translated into Chinese [31]. It consists of 3 subscales, which is professional cognition (3 items), professional impact (3 items), and professional evaluation (4 items), with the responses to each item provided on a 5-point Likert scale (1 = never to 5 = very often). The total score of the scale ranged from 10 to 50. A higher average score on the scale indicates a stronger professional identity of nursing interns [31]. The Cronbach’s α coefficient of the scale was 0.710 for the overall version and 0.820 for the Chinese version, the Cronbach alpha coefficient in this study was 0.898.

#### Nursing students’ perception of hospital caring environment scale

Nursing interns’ perception of climate of caring was measured by The Nursing Students’ Perception of Hospital Caring Environment Scale [32], which includes the following 4 aspects: clinical interns’ perception of patient care given by the teaching teacher (17 items), perception of the care given by the clinical teaching teacher (19items), perception of care among peers (14 items), and self-care given by other medical staff in the department (8 items). The items are evaluated on a 5-point scale ranging from 1 (completely disagree) to 5 (completely agree), with higher scores indicating higher percept hospital caring environment [32], the Cronbach alpha coefficient was 0.976 for the total scale, and 0.960 in this study.

### Ethical consideration

The Ethics Committee of Xiangya School of nursing, Central South University approved the study protocol (Approval No. E2022213). First, we consulted with the nursing department in the hospital for permission of this study. Then, an online questionnaire was sent to a chat group that included all nursing interns from various schools. The purpose of this survey was written on the first page of the questionnaire for respondents, and respondents digitally signed a written informed consent form. Participants were also informed that they had the option to withdraw from the study at any time.

### Data analysis

The SPSS 28.0 software program (IBM Corp.) was used for data analysis. Firstly, the normality of the data was tested using the skewness statistic and normal probability plot. Continuous variables with normal distribution were expressed as mean ± standard deviation (SD), whereas non-normally distributed variables were expressed as reported using the median and quartile (*P*_25_, *P*_75_). Categorical data were presented as frequencies and percentages. Given the skewed distribution of the variables in this study, the Mann-Whitney *U* test and Kruskal-Wallis *H* test were used to compare the differences in the transition shock scores of nursing interns with different demographic characteristics. Subsequently, the correlations between transition shock, patient safety attitudes, professional identity and climate of caring were assessed using the *Spearman* correlation method. Finally, multiple linear regression was used to analyzed the factors influencing the transition shock, after ensuring that the residual of the dependent variable fulfil the four requirements of ‘linearity’, ‘independence’, ‘homogeneity of variance’ and ‘normal distribution’. The level of acceptable significance was set at *p* < 0.05.

## Results

### Participants’ characteristics

A total of 356 valid questionnaires were collected with most respondents being female (*n* = 294, 82.6%). The mean age of the participants was 21.11 years (SD = 1.14 years) ranging from 18 to 24 years. Of the nursing interns surveyed in this study, 178 (50%) had chosen nursing as their first major and 294 (82.6%) nursing interns reported that their health situation were great. Furthermore, 215(60.5%) were pursuing a bachelor’s degree, a majority of nursing interns 243 (68.3%) reported that they like nursing profession. Table 1 shown the characteristics of nursing interns and their scores on transition shock based on different characteristics. The reason for choose major nursing (*P* < 0.01), whether there are nurses among relatives (*P* < 0.001), body health situation (*P* < 0.001), before nursing internship whether want be a nurse (*P* < 0.001), after nursing internship whether want be a nurse (*P* < 0.001), whether like nursing profession (*P* < 0.001), these different demography characteristics shown statistical differences on nursing interns’ transition shock scores.

**Table 1:** Comparison of the scores of nursing interns’ transition shock in different demography characteristics (*n* = 356)

		*n*	%	Transition shock		
				*Median (Q25, Q75)*	*Z/H*	*P*
**Gender**					-2.26^a^	0.023
	Male	62	17.4	2.16(1.93,2.50)		
	Female	294	82.6	2.30(2.11,2.55)		
**Age** (Year)					0.8^b^	0.67
	18–20	112	31.5	2.27(2.05,2.50)		
	20–22	219	61.5	2.27(2.11,2.55)		
	≥ 23	25	7	2.33(1.83,2.72)		
**Education level**					-2.98^a^	0.003
	Associate	141	39.6	2.22(2.0,2.44)		
	Bachelor	215	60.4	2.33(2.11,2.61)		
**The reason for choose major nursing**					24.91^b^	<0.001
	Self decision	178	50	2.16 (2.00,2.44)		
	School adjustment	83	23.3	2.44(2.16,2.72)		
	Parents request	64	18	2.38 (2.16,2.59)		
	Others recommendation	31	8.7	2.33 (2.22,2.55)		
**Whether there are nurses among relatives?**					-3.39^a^	<0.001
	No	234	65.7	2.33(2.11,2.55)		
	Yes	122	34.3	2.22(1.94,2.45)		
**How do you feel about your body health?**					21.11^b^	<0.001
	Great	294	82.6	2.27(2.05,2.50)		
	General	59	16.6	2.50(2.22,2.77)		
	Bad	2	0.6	2.27(2.22,2.27)		
	Unclear	1	0.3	2.72(2.72,2.72)		
**Before nursing internship, do you want be a nurse?**					15.64^b^	<0.001
	Yes	259	72.8	2.22(2.00,2.50)		
	No	23	6.5	2.50(2.27,2.83)		
	Unclear	74	20.8	2.33(2.16,2.55)		
**After nursing internship, do you want be a nurse?**					41.73^b^	<0.001
	Yes	256	71.9	2.22(2.00,2.44)		
	No	28	7.9	2.61(2.44,2.97)		
	Unclear	72	20.2	2.46(2.22,2.66)		
**Whether like nursing profession?**					52.75^b^	<0.001
	Yes	243	68.3	2.22(2.00, 2.38)		
	No	38	10.7	2.55(2.208,2.88)		
	Unclear	75	21.1	2.50(2.27,2.66)		

Note: ^a^: Mann-Whitney U test; ^b^: Kruskal-Wallis H test

### The scores of transition shock, patient safety attitudes, professional identity, and climate of caring among nursing interns

Given the skewed distribution of the variables, median and quartile (P25, P75) were used to describe the results. Standardization and comparability among subscales were ensured through the formula: the score of subscale/scale = the sum score of items ÷ the number of items.

As shown in Table 2, transition shock among nursing interns scored 2.27 (2.05, 2.50) at a moderate level, and the overwhelming practicum workload scored 2.66 (2.33, 3.00), which was higher than other subscales, followed by conflict between theory and practice 2.33 (2.00,2.66). The patient safety attitudes of nursing interns scored 4.15 (3.78,4.60), and the teamwork culture scored 5.0 (4.16,5.00), which was higher than other subscales. The professional identity scored 3.90 (3.40,4.30) and the climate of caring scored 4.55 (4.00,4.94) among nursing interns.

**Table 2:** The scores of transition shock, patient safety attitudes, professional identity, and climate of caring among nursing interns(*n* = 356)

Variable	Domain	Min	Max	Median (Q25, Q75)
Transition shock	Conflict between theory and practice	1	4	2.33(2.00, 2.66)
	Overwhelming practicum workload	1	4	2.66(2.33,3.00)
	Loss of social support	1	4	2.0(2.0.,2.50)
	Shrinking interpersonal relationship	1	4	2.0(2.0,2.66)
	Confusion in professional nursing values	1	4	2.20(2.00,2.60)
	Incongruity in clinical practicum and personal lifes	1	4	2.0(2.00,2.00)
	Over all	1	3.78	2.27(2.05,2.50)
Climate of caring	One-to-one nursing teacher’s caring for patients	1.76	5	4.64(4.00,4.94)
	One-to-one nursing teacher’s caring for students	1.58	5	4.73(4.00,5.00)
	The peers in hospital	1	5	4.50(4.00,5.00))
	The other medical workers in wards	1.75	5	4.25(4.00,5.00)
	Over all	1.52	5	4.55(4.00,4.94)
Professional identity	Professional cognition	1	5	3.66(3.33,4.00)
	Professional evaluation	1.33	5	4.0(3.33,4.33)
	Professional impact	1.75	5	3.87(3.25,4.25)
	Over all	1.9	5	3.90(3.40,4.30)
Patient safety attitudes	Safety culture	0.5	5	4.87(4.00,5.00)
	Teamwork culture	0	5	5.0(4.16,5.00)
	Error disclosure culture	0	5	4.20(3.40,4.80)
	Implement security conditions	0	5	4.75(4.00,5.00)
	Over all	1.09	5	4.60(4.00,4.91)

### Correlation analysis of transition shock, patient safety attitudes, professional identity, and climate of caring among nursing interns

As shown in Table 3, nursing interns’ transition shock had statistically significant negative correlation with patient safety attitudes (*r*=-0.386, *p* < 0.01), professional identity (*r*=-0.531, *p* < 0.01), and climate of caring (*r*=-0.447, *p* < 0.01).

**Table 3:** Correlation analysis of transition shock, patient safety attitudes, professional identity, and climate of caring among nursing interns(*n* = 356)

**Variable**	**Domain**	Transition shock
		*Spearman correction coefficients(r)*
Climate of caring	One-to-one nursing teacher’s caring for patients	− 0.429**
	One-to-one nursing teacher’s caring for students	− 0.422**
	The peers in hospital	− 0.359**
	The other medical workers in wards	− 0.411**
	Over all	− 0.447**
Patient safety attitudes	Safety culture	− 0.362**
	Teamwork culture	− 0.396**
	Error disclosure culture	− 0.265**
	Implement security conditions	− 0.347**
	Over all	− 0.386**
Professional identity	Professional cognition	− 0.396**
	Professional evaluation	− 0.523**
	Professional impact	− 0.515**
	Over all	− 0.531**

**<0.01

### Multiple linear regression analysis on factors influencing nursing interns’ transition shock

Table 4 presented the multiple linear regression of influencing factors on the transition shock among nursing interns, the results indicated the reason for major nursing (β = 0.099, *P* = 0.021), Whether there are nurses among relatives (β=-0.119, *P* = 0.005), Whether like nursing profession (β = 0.094, *P* = 0.043), patient safety attitudes (β=-0.135, *P* = 0.003), professional identity (β=-0.369, *P* < 0.001), and climate of caring (β=-0.190, *P* < 0.001) were significantly predicted transition shock of nursing interns and explained 39.3% of the variance (*F* = 37.596, *R*^2^ = 0.393, δ*R*^2^ = 0.382, *p* < 0.001).

**Table 4:** Multiple linear stepwise regression analysis on influencing factors of transition shock among nursing interns(*n* = 356)

Model	Coefficient^a^ Unstandardized coefficient		Standard coefficient	*t*	*P*
	*B*	*SE*	*Beta*		
(Constant)^a^	4.202	0.195		21.588	< 0.001
The reason for major nursing^b^	0.042	0.018	0.099	2.324	0.021
Whether there are nurses among relatives?^c^	-0.106	0.037	-0.119	-2.831	0.005
Whether like nursing profession?^d^	0.048	0.024	0.094	2.035	0.043
Climate of caring	-0.143	0.035	-0.190	-4.062	< 0.001
Patient safety attitudes	-0.080	0.027	-0.135	-3.007	0.003
Professional identity	-0.247	0.033	-0.369	-7.449	< 0.001

Note:

^a^ Dependent variable: transition shock

^b^ Scores of the reason for choose major nursing: self decision = 1, school adjustment = 2, parents request = 3, others recommendation = 4

^c^ Scores of whether there are nurses among relatives: yes = 1, no = 2

^d^ Scores of whether like nursing profession: yes = 1, no = 2

## Disscusion

The study found that nursing interns experienced a moderate level of transition shock and its negative correlation with patient safety attitudes, professional identity and perceptive climate of caring. These findings provide valuable insights for nursing managers and educators, which can inform the development of nursing internship programs that facilitate seamless transitions to practice for nursing interns. By fostering these factors, nursing interns can adeptly assume clinical nursing roles and contribute to delivering high-quality, safe nursing care for patients.

This study demonstrated that interns experienced a moderate level of transition shock. The highest contributing factor was the overwhelming practicum workloads, followed by conflicts between theoretical knowledge and practical application. These results align with studies [33, 34] which identified heavy physical workload as a major contributor to a moderate level of interns’ transition shock. The shortage of nurses has become a pressing issue in China. Nursing managers, expecting interns to perform clinical practices as skilled nurses. Additionally, previous study [35] indicated nursing interns were exploited by certain staff members and assigned tasks unrelated to nursing or beyond the scope of an internship. Those increased the workload and transition shock experienced by nursing interns. Moreover, the conflict between theory and practice, making it challenging for nursing interns to address patients’ problems in clinical settings which has consistent with previous study [36]. Nursing interns often assume the role of passive recipients of knowledge during their education. However, clinical practice demands not only the acquisition of essential nursing practice skills but also the development of professional critical thinking abilities. Lack of professional skills and limited clinical practice knowledge could negatively impact interns’ self-confidence in necessary nursing competencies thus result in transition shock. It is recommended that nursing managers and educators promote a supportive organizational culture, providing structured support to nursing interns by enhancing their clinical knowledge and addressing their negative emotions to ease transition shock. Within this supportive environment, nursing managers can consider reducing interns’ workloads, implementing flexible work schedules and workload adjustments tailored to the characteristics of interns. Nursing educators should recognize the occurrence of transition shock among interns, comprehend the specific causes, and implement support strategies to bolster their confidence. Moreover, nursing units could support standardized, unit-specific training in professional skills, which can boost interns’ enthusiasm for their work and facilitate a smooth transition.

Our results showed a negative correlation between patient safety attitudes and transition shock in nursing interns. Previous studies [37, 38] has highlighted that interns often struggle to identify various clinical symptoms in patients during transition periods, leading to stress and lack of confidence, which undermines their faith in providing safe care. The teamwork culture subscale was determined to be the most significantly associated with interns’ transition shock in this study. Previous research [39] have explored the characteristics of an exceptional nursing teamwork culture was mutual understanding and effective communication. This study highlights the crucial role of teamwork culture in enhancing patient safety attitudes and reducing transition levels among nursing interns. Nurse educators and managers could create a teamwork culture that emphasized communication and collaboration among nursing unit. Holding regular meetings to discuss safety nursing, address concerns, and share ideas, encourage respectful communication between nursing interns and senior nurses. Organizing group activities among nursing unit that enhance nursing teams’ problem-solving and decision-making skills further strengthens teamwork culture. Additionally, involving interns in patient care evaluation and nursing plan design and encouraging them to contribute their expertise.

The professional identity also shown statistically significant negative correlation with transition shock among nursing interns. Interns with higher professional identity values experience lower transition shock levels [16]. Professional identity, as an aspect of self-concept, which can aid interns in their successful transition into practice [40]. Therefore, it is highly recommended that nursing schools and clinical nursing departments collaborate to create a robust and comprehensive career planning curriculum for nursing interns, aim to enhancing their professional identity [4, 41]. This can be achieved by offering interns diverse opportunities to be exposed to various nursing specialties, clinical settings, and patient populations, which will help them to identify areas they are passionate about and expand their understanding of the profession. Integrate theory with practice, nursing schools should provide more opportunities for interns to interact with actual patients in clinical settings, rather than simply having contact with standardized patients in simulated hospitals. A combination of classroom-based education and hands-on training is necessary to ensure that interns develop a broad understanding of nursing concepts and real-world experiences, and enable interns to develop soft skills such as communication, leadership, and critical thinking. These approaches above will foster the development of professional identity, paving the way for interns’ successful transition into practice.

Our findings demonstrated climate of caring in hospital help reduce nursing interns’ level of transition shock. A perceived climate of caring fosters interns’ senses of belonging and recognition in hospital [42, 43], thus mitigate transition shock. Interestingly, the one-to-one nursing teacher’s caring for patient subscale has strongest inverse relationship with transition shock among nursing interns. The daily provision of compassionate nursing care by teachers greatly influences interns [44], as it strengthens their professional identity, promotes a sense of meaning in their work, and facilitates acceptance of their professional roles. This result highlights the critical role mentors play in exemplifying professional behavior, empathy, and compassion for interns. By sharing their own professional experiences, challenges, and growth, mentors can effectively convey the importance of empathy and compassion in nursing practice to interns. In conclusion, our study emphasizes the necessary for nurse managers and educators to create a climate of caring within nursing units, which exemplifies professional caring behavior, accentuates caring values, encourages a teamwork culture, and offers structured support. Nurse managers and educators should demonstrate caring behaviors towards patients, colleagues, and interns, setting an example for team.

## Limitation

This study had several limitations that should be considered. Firstly, the cross-sectional design of the study prevents making causal inferences. Secondly, the use of convenience sampling may limit the generalizability of the results. Lastly, the reliance on self-reported questionnaires may not fully capture the nursing interns’ transition shock experience. Therefore, future research should aim to address these limitations by using larger and more diverse samples, and conducting more in-depth data analyses to further explore the mechanisms of impact, which provide specific targets for interventions to mitigate the level of transition shock experienced by interns. Additionally, employing an interview methodology would offer greater opportunities to follow up on nursing interns’ thoughts and specific needs related to this phenomenon, providing a more comprehensive understanding of their experiences.

## Implication

This study results reveal the presence of a moderate level of transition shock among nursing interns, underscoring the imperative for nursing administrators and educators to emphasize this phenomenon. Additionally, this study contributes to enriching the theoretical foundation for future interventions aimed at enhancing the practical experience of nursing interns. Our correlation analysis has demonstrated a negative association between transition shock and professional identity, patient safety attitudes, and the caring climate, indicating the need for interventions to bolster these aspects and improve the internship experience. Therefore, it is essential for nursing managers and educators to prioritize the establishment of a supportive and caring nursing units’ environment for nursing interns. This involves providing mentorship, delivering patient safety-focused education, and fostering the development of professional identity, which has far-reaching implications for the overall well-being of nursing interns and the quality of nursing care.

## Conclusion

Nursing interns experience transition shock at a moderate level, which is negatively associated with patient safety attitudes, professional identity and climate of caring. Our study presents valuable insights for nurse managers and educators, aimed at facilitating successful transitions for nursing interns. To enhance professional identity and promote successful transition, we recommend the development of a comprehensive career planning curriculum by educators and nursing departments. The training content should focus on broadening the developmental dimensions of nursing interns, bridging the gap between theoretical knowledge and practical application, providing interns ample opportunities for independent learning in patient care. Furthermore, establishing climate of caring within the hospital is crucial in providing nursing interns with the necessary organizational support and teamwork culture, which fosters the competence and confidence needed to establish patient safety attitudes, deliver high-quality nursing care and actively engage in clinical practice. And, this climate also emphasizing communication and collaboration between nursing interns and senior nurses, which is vital to promoting professional growth, alleviating negative emotions, then guiding a smooth transition.

## Data Availability

The datasets generated and/or analysed during the present study are not publicly available due to the data being proprietary and confidential records of The Third Xiangya Hospital / Xiangya School of Nursing, Central South University, but are available from the corresponding author on reasonable request.

## References

[1] Antonello F, Antonello M (2020). [Clinical rationale training and nursing internship]. Rev Infirm.

[2] Regulations on Nurses (promulgated by Decree No. 517 of the State Council of the People’s Republic of China on 31 January 2008)(Revised in accordance with the Decision of the State Council on Amending and Abolishing Some Administrative Regulations of 27 March 2020).

[3] Haleem DM, Manetti W, Evanina K, Gallagher R (2011). A senior internship: facilitating the transition to nursing practice. Nurse Educ.

[4] Hampton KB, Smeltzer SC, Ross JG (2021). The transition from nursing student to practicing nurse: an integrative review of transition to practice programs. Nurse Educ Pract.

[5] Duchscher JE (2009). Transition shock: the initial stage of role adaptation for newly graduated registered nurses. J Adv Nurs.

[6] Weller-Newton JM, Murray M, Phillips C, Laging B, McGillion A (2022). Transition to Practice Programs in nursing: a Rapid Review. J Contin Educ Nurs.

[7] Ko YJ, Kim SY. Transition shock experience of nursing students in clinical practice: a Phenomenological Approach. Healthc (Basel) 2022, 10(4). 10.3390/healthcare10040613 PMC9029544 35455791

[8] Cao X, Li J, Gong S (2021). Effects of resilience, social support, and work environment on turnover intention in newly graduated nurses: the mediating role of transition shock. J Nurs Manag.

[9] Kim EY, Yeo JH (2021). Transition shock and job satisfaction changes among newly graduated nurses in their first year of work: a prospective longitudinal study. J Nurs Manag.

[10] Labrague LJ, De Los Santos JAA (2020). Transition shock and newly graduated nurses’ job outcomes and select patient outcomes: a cross-sectional study. J Nurs Manag.

[11] Hongzhen Z, He J (2017). Study on the status of nursing practical students’ patient safety attitudes and professionalism. Chin J Nurs Educ.

[12] Murray M, Sundin D, Cope V (2019). New graduate nurses’ understanding and attitudes about patient safety upon transition to practice. J Clin Nurs.

[13] Rahmani P, Molaei Tavani F, Sheikhalipour Z, Behshid M, Khodayari MT, Zadi Akhuleh O (2022). The relationship between attitude of nurses toward the patient safety and missed nursing care: a predictive study. J Healthc Qual Res.

[14] Kelly J, Watson R, Watson J, Needham M, Driscoll LO (2017). Studying the old masters of nursing: a critical student experience for developing nursing identity. Nurse Educ Pract.

[15] Arreciado Marañón A, Isla Pera MP (2015). Theory and practice in the construction of professional identity in nursing students: a qualitative study. Nurse Educ Today.

[16] Sun L, Gao Y, Yang J, Zang XY, Wang YG (2016). The impact of professional identity on role stress in nursing students: a cross-sectional study. Int J Nurs Stud.

[17] Heldal F, Kongsvik T, Håland E (2019). Advancing the status of nursing: reconstructing professional nursing identity through patient safety work. BMC Health Serv Res.

[18] Wenxia Z, Feifei C, Min H, Li C, Aihong L, Xingfeng L (2022). The status and associated factors of junior nurses’ transition shock: a cross-sectional study. J Nurs Adm Manag.

[19] Cai T (2021). Transition of newly graduated nurses in China: an evaluation study. Nurse Educ Pract.

[20] Hughes L (1992). Faculty-student interactions and the student-perceived climate for caring. ANS Adv Nurs Sci.

[21] Rathert C, Ishqaidef G, Porter TH. Caring work environments and clinician emotional exhaustion: empirical test of an exploratory model. Health Care Manage Rev 2022, 47(1). 10.1097/HMR.0000000000000294 33298806

[22] Fu W, Deshpande SP (2014). The impact of caring climate, job satisfaction, and Organizational Commitment on Job performance of employees in a China’s Insurance Company. J Bus Ethics.

[23] Romero-Martín M, Safont-Montes JC, Robles-Romero JM, Jiménez-Picón N, da Costa E, Gómez-Salgado J (2022). Caring behaviours demonstrated to nursing students in the interpersonal relation with the faculty: a cross sectional study. Nurse Educ Today.

[24] Alan Stuart KO (1995). Kendall’s advanced theory of statistics. J Am Stat Assoc.

[25] Kim S, Shin YS (2019). Validity and reliability of the transition shock scale for undergraduate nursing students. J Korean Acad Soc Nurs Educ.

[26] Yu xuanH, Shao yu M, Jia lu Z (2021). Ya Ping B, Qiong yue Z: the reliability and validity of the Chinese version of the transition shock scale for undergraduate nursing students. China J Nurse Eduction.

[27] Yu xuan H. Study on the degree of transition shock and its influencing factors among undergraduate nursing students. *Master1027674/d.cnki.gcyku.2022.001631*. Chongqing Medical University,; 2022.

[28] Liao JM, Etchegaray JM, Williams ST, Berger DH, Bell SK, Thomas EJ (2014). Assessing medical students’ perceptions of patient safety: the medical student safety attitudes and professionalism survey. Acad Med.

[29] Hongzhen Z, He J (2016). Reliability and validity of a Chinese version of the nursing student patient safety attitudes and professionalism survey. Chin Nurs Manage.

[30] Brown R, Condor S, Mathews A, Wade G, Williams J (1986). Explaining intergroup differentiation in an industrial organization. J Occup Psychol.

[31] Lu H, While AE, Barriball KL (2007). Job satisfaction and its related factors: a questionnaire survey of hospital nurses in Mainland China. Int J Nurs Stud.

[32] Li C. Survey on nursing students’ perception of caring climate in Hospital. *Master* Huazhong University of Science and Technology; 2011.

[33] Su Q, Jiang M, Yun B, Ma Y, Zuo Y, Han L (2021). Effect of clinical teaching behaviours on transition shock in graduate nurses. J Adv Nurs.

[34] Lian, Ying (2020). Caimei Z: correlation between nursing interns’ transformation impact and feedback seeking behavior. J Nurs Sci.

[35] Bahari G, Alharbi F, Alharbi O (2022). Facilitators of and barriers to success in nursing internship programs: a qualitative study of interns’ and faculty members’ perspectives. Nurse Educ Today.

[36] Nour V, Williams AM (2019). Theory becoming Alive: the learning transition process of newly graduated nurses in Canada. Can J Nurs Res.

[37] Herron EK (2018). New graduate nurses’ preparation for recognition and prevention of failure to rescue: a qualitative study. J Clin Nurs.

[38] Lee JE, Sim IO (2020). Gap between college education and clinical practice: experience of newly graduated nurses. Nurs Open.

[39] Keats JP (2019). Leadership and Teamwork: essential roles in Patient Safety. Obstet Gynecol Clin North Am.

[40] Su Q, Yun B, Yuet Foon Chung L, Chen L, Zuo Y, Liu J, Han L (2021). Clinical teaching behaviour effects professional identity and transition shock in new nurses in western China: a cross-sectional study. Nurs Open.

[41] Graf AC, Jacob E, Twigg D, Nattabi B (2020). Contemporary nursing graduates’ transition to practice: a critical review of transition models. J Clin Nurs.

[42] Letcher DC, Nelson ML (2014). Creating a culture of caring: a partnership bundle. J Nurs Adm.

[43] Inocian EP, Hill MB, Felicilda-Reynaldo RFD, Kelly SH, Paragas ED, Turk MT (2022). Factors in the clinical learning environment that influence caring behaviors of undergraduate nursing students: an integrative review. Nurse Educ Pract.

[44] Chen F, Liu Y, Wang X, Dong H (2021). Transition shock, preceptor support and nursing competency among newly graduated registered nurses: a cross-sectional study. Nurse Educ Today.

